# Triboelectric nanogenerator based on Teflon/vitamin B1 powder for self-powered humidity sensing

**DOI:** 10.3762/bjnano.11.123

**Published:** 2020-09-11

**Authors:** Liangyi Zhang, Huan Li, Yiyuan Xie, Jing Guo, Zhiyuan Zhu

**Affiliations:** 1 Chongqing Key Laboratory of Nonlinear Circuits and Intelligent Information Processing, College of Electronic and Information Engineering, Southwest University, Chongqing, China; 2 Key Laboratory of Networks and Cloud Computing Security of Universities in Chongqing, College of Electronic and Information Engineering, Southwest University, Chongqing, China; 3 Ocean College, Zhejiang University, Zhejiang, China

**Keywords:** humidity sensor, self-powered system, triboelectric nanogenerators (TENGs), triboelectrification, vitamin B1

## Abstract

Recently, there has been growing interest in triboelectric nanogenerators (TENGs) that can effectively convert various forms of mechanical energy input into electrical energy. In the present study, a novel Teflon/vitamin B1 powder based triboelectric nanogenerator (TVB-TENG) is proposed. Paper is utilized as a supporting platform for triboelectrification between a commercial Teflon tape and vitamin B1 powder. The measured open-circuit voltage was approximately 340 V. The TVB-TENG can be applied as a humidity sensor and exhibits a linear and reversible response to the relative humidity of the environment. Moreover, the change in relative humidity is also indicated by the change in luminosity of a set of light-emitting diodes (LEDs) integrated in the TVB-TENG system. The TVB-TENG proposed in this study illustrates a cost-effective method for portable power supply and sensing devices.

## Introduction

Recently, there has been unprecedented advancement in the internet of things (IoT) technology, which includes environmental monitoring and intelligent community applications. Particularly, humidity sensing has been investigated in environmental monitoring, and in other sectors, such as agriculture, food safety, wearable electronics, and wireless sensor networks [1–4]. However, conventional power generation is needed to supply energy to these sensor networks, which leads to increased energy usage and adverse impacts on the environment. More specifically, the degradation of the urban environment has been increasing due to the current life habits of the population [5–7]. Moreover, a variety of sensors are often placed in severe environmental conditions, which might restrict their power supply options [8–12]. As a result, several lines of research have been focused on the development of methods for harvesting energy from the surrounding environment and converting it into electrical power. Through the design of portable electronics and wireless sensor systems [13–16], which harvest energy from the environment, the adverse environmental effects caused by battery-powered systems can be mitigated [17–21]. Hence, the investigation of self-powered sensors which harvest energy from the surrounding environment is highly sustainable.

Triboelectric nanogenerators (TENGs) have been growing in popularity for use as a novel technology to harvest energy. TENGs have a significant impact on the advancement of wearable electronics, intelligent robots, and the IoT [22–28]. Presently, TENGs are used to harvest various forms of mechanical energy from the surrounding environment, such as acoustic energy, wind, vibrations and human motion [29–33]. Recently, TENG-based sensors have attracted increased attention [34–41]. In 2014, Ga-doped ZnO was used for the fabrication of piezo-humidity sensors with a high sensitivity and a fast response [42]. In 2018, Vivekananthan et al. proposed sustainable energy harvesting and battery-free humidity sensors by using biocompatible collagen nanofibrils [43]. More recently, Zhang et al. developed a TENG-driven self-powered flexible humidity sensor based on a tin disulfide nanoflower/reduced graphene oxide (SnS_2_/rGO) hybrid nanomaterial [44]. However, the large-scale application of TENGs for humidity sensing is hindered by the high costs involved in their complex production and the high facility costs for particular manufacturing process. Thus, intensive research is required for the design of TENGs based on commercially available, cost-effective and feasible materials. Furthermore, TENGs should be seamlessly integrated and fabricated using a simple process, particularly regarding multifunctional sensing applications.

In the present study, a novel Teflon/vitamin B1 powder based triboelectric nanogenerator (TVB-TENG) is proposed. Vitamin B1 is an essential water-soluble vitamin which stays in the human body for only a few hours. Vitamin B1 is a coenzyme involved in the metabolism of sugar, protein and fat, which is found in grains, beans, pork, and other sources. Since it is cost-effective, environmentally friendly, non-poisonous and soluble, vitamin B1 can be used as a lubricant to make a friction nanogenerator. Due to its sustainability and flexibility, paper can be used as a substrate and supporting structure. The conductive electrode is made of copper foil, while the triboelectric pair is comprised of Teflon tape and vitamin B1 powder. The approximate values of the output power density of the TVB-TENG can reach 120.13 µW/cm^2^. In addition, the approximate values of the open-circuit voltage (*V*_oc_) and short-circuit current (*I*_sc_) of this device were 340 V and 46.3 μA, respectively. These results indicate that sufficient power can be supplied to systems with low power requirements. As an extension of previous work [45], the self-powered TVB-TENG designed in this study can be used to measure the relative humidity and exhibits good linearity and reversibility. In addition, the materials used in the manufacturing of the TENGs are nontoxic and degradable. Furthermore, light-emitting diodes (LEDs) are integrated with the humidity sensor and the luminosity is an indicator of the relative humidity (RH) from the surrounding environment.

## Experimental

**Figure 1:**
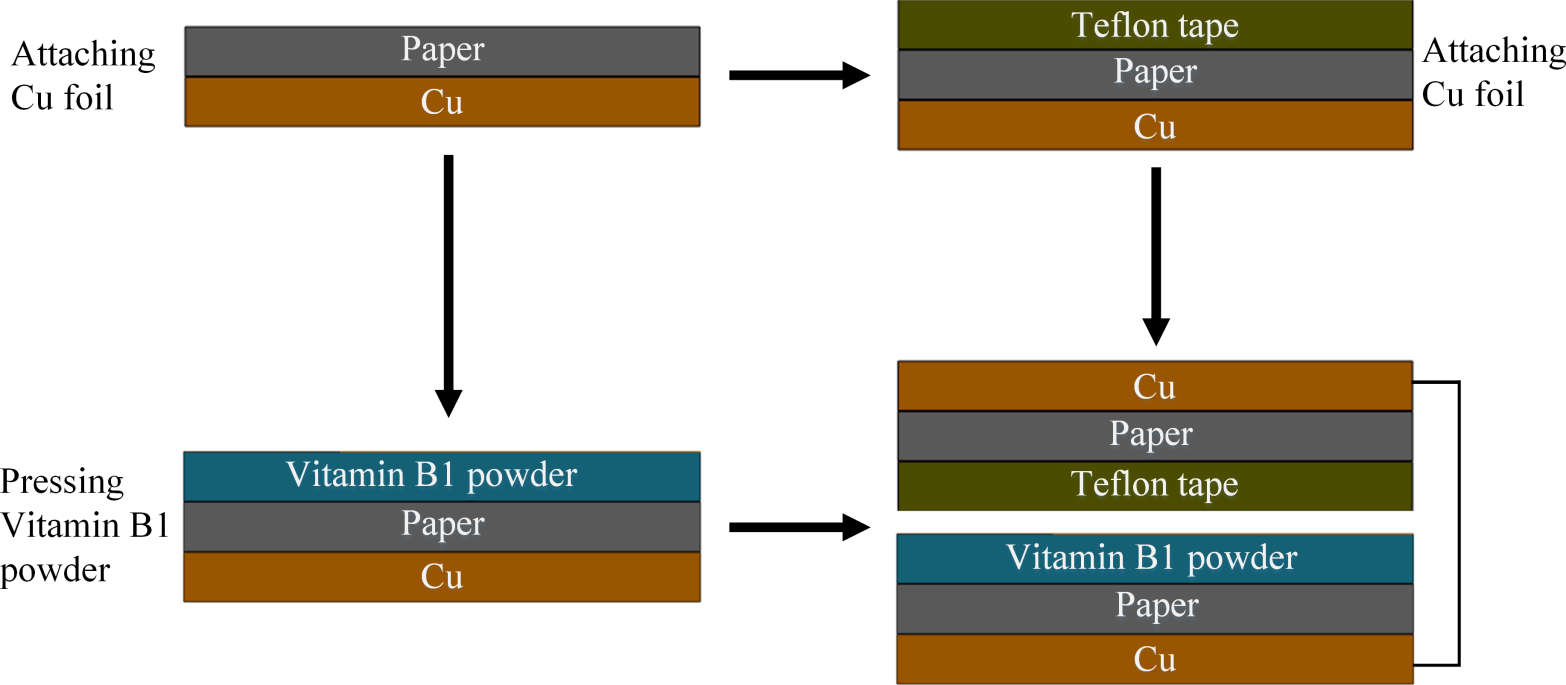
The manufacturing process of the TVB-TENG structure.

The conductive copper foil tape is used as the electrode, while the paper (manufacturer: Miao Ben) at the bottom serves as a supporting platform. The Teflon tape (manufacturer: RUIGUAN) is cut to the desired size (4 cm × 5 cm) and pasted to the bottom of the conductive copper foil tape, serving as one element of the triboelectric pair. In addition, ≈20 ± 5 mg of vitamin B1 powder (manufacturer: Huazhong Pharmaceutical Co. LTD) is pressed against the bottom of another conductive copper foil tape with double-sided tape, working as the other element in the triboelectric pair. The fabrication process of the TVB-TENG proposed in this study is illustrated in Figure 1 and the actual price of the material used to make the TENG is only approximately CN¥ 0.5. An oscilloscope (probe: 100 MΩ) was used to measure the voltage across the external load (a variable resistor) and a high-precision multifunctional electronic scale, with an accuracy of 0.001 g, was used to measure the required amount of vitamin B1 powder.

## Results and Discussion

**Figure 2:**
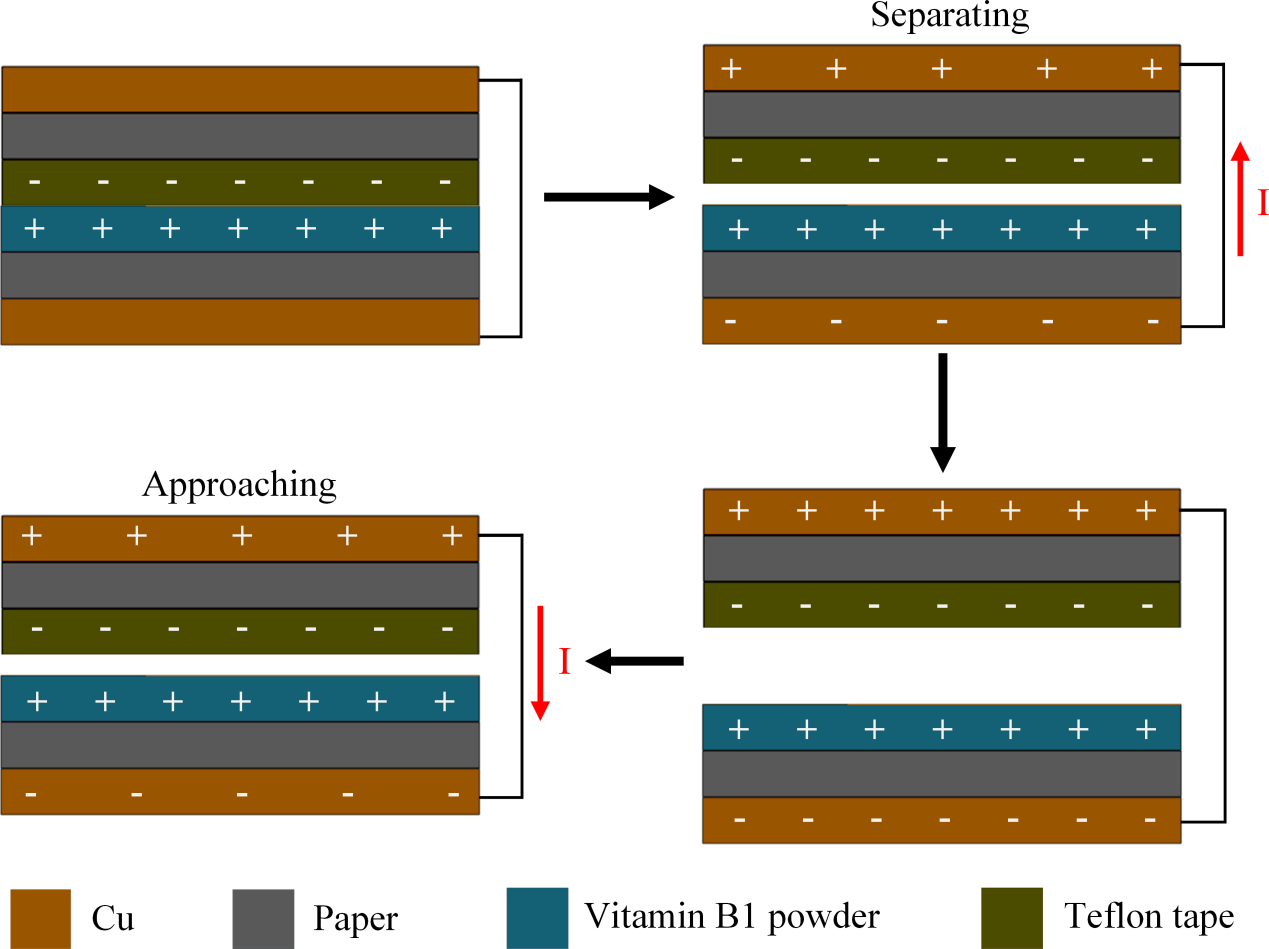
The working mechanism of the TVB-TENG structure.

As shown in Figure 2, the working mechanism of TVB-TENG is based on contact triboelectrification and electrostatic induction. First, when the device is externally compressed, electrons are transferred from the B1 vitamin membrane to the Teflon membrane. The contact surface between the vitamin B1 membrane and the Teflon membrane is separated in the absence of an external force. There is a positive charge transfer from the conductive copper foil tape at the bottom of the TVB-TENG, to the conductive copper foil tape at the top, leading to an electric field equilibrium due to electrostatic induction. As a result, a potential difference between the electrodes is generated. Subsequently, when the TVB-TENG is pressed again, an opposite potential difference is produced due to the triboelectrification principle. As such, there is a positive charge transfer from the top of the conductive copper foil tape of the TVB-TENG to the bottom one [46]. Therefore, it is expected that TVB-TENGs can produce a stable output power under the sustained effect of an external force. The potential distribution is illustrated to obtain a comprehensive understanding of this phenomenon.

**Figure 3:**
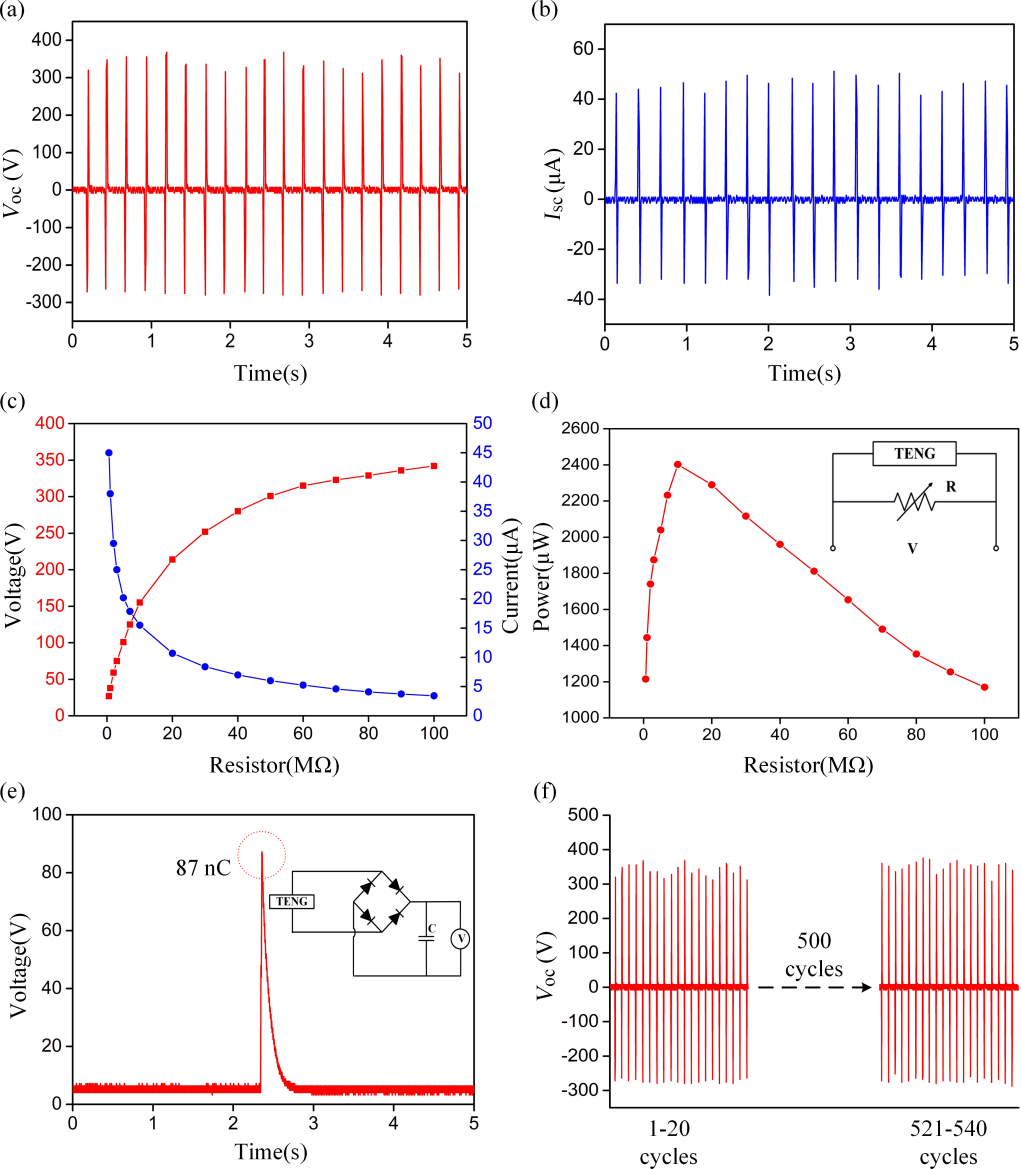
(a) The output voltage (matched load of 100 MΩ) and (b) short-circuit current (matched load of 100 kΩ) in a TENG. (c) The behavior of the output voltage and short-circuit current upon changing the load resistance. (d) The behavior of the output power density upon changing the load resistance. (e) TVB-TENG with a full-wave bridge rectifier for charging a 1 nF capacitor. In one cycle, 87 nC of charge is transferred. (f) The reliability of TVB-TENG was studied over 500 working cycles.

An oscilloscope (probe: 100 MΩ) was used to measure the voltage across the external load (a variable resistor). The output current was calculated by using the measured voltage and the load resistance. As illustrated in Figure 3a and Figure 3b, the output voltage and the current reached a peak at 340 V and 46.3 μA, corresponding to a load resistance of 100 MΩ and 100 kΩ, respectively. As shown in Figure 3c, when the load resistance was increased from 100 kΩ to 100 MΩ, the measured output voltage showed an increasing trend. As shown in Figure 3d, the output power reached its peak at 2402.5 μW at a loading resistance of 10 MΩ. Accordingly, the fabricated internal resistance of the TENGs was close to 10 MΩ. Consequently, considering the size of the fabricated TENG (4 cm × 5 cm), the maximum power density is 120.13 μW/cm^2^. Since an external resistance of 100 MΩ is much higher than 10 MΩ (roughly equal to the internal resistance), the output voltage (at a 100 MΩ load) is approximated to be equal to the open circuit voltage. Similarly, for a load of 100 kΩ, the corresponding output current can be considered to be equal to the short circuit current. In addition, as shown in Figure 3e, the charging capacity of the prepared TVB-TENG was investigated by integrating a full-wave rectifier bridge to charge a 1 nF capacitor. The maximum capacitive voltage was 87 V, and in one cycle, a charge of 87 nC was transferred. The reliability of the manufactured TVB-TENG was also investigated. As shown in Figure 3f, the output voltage of the TENG remains steady even after 500 external force testing cycles.

**Figure 4:**
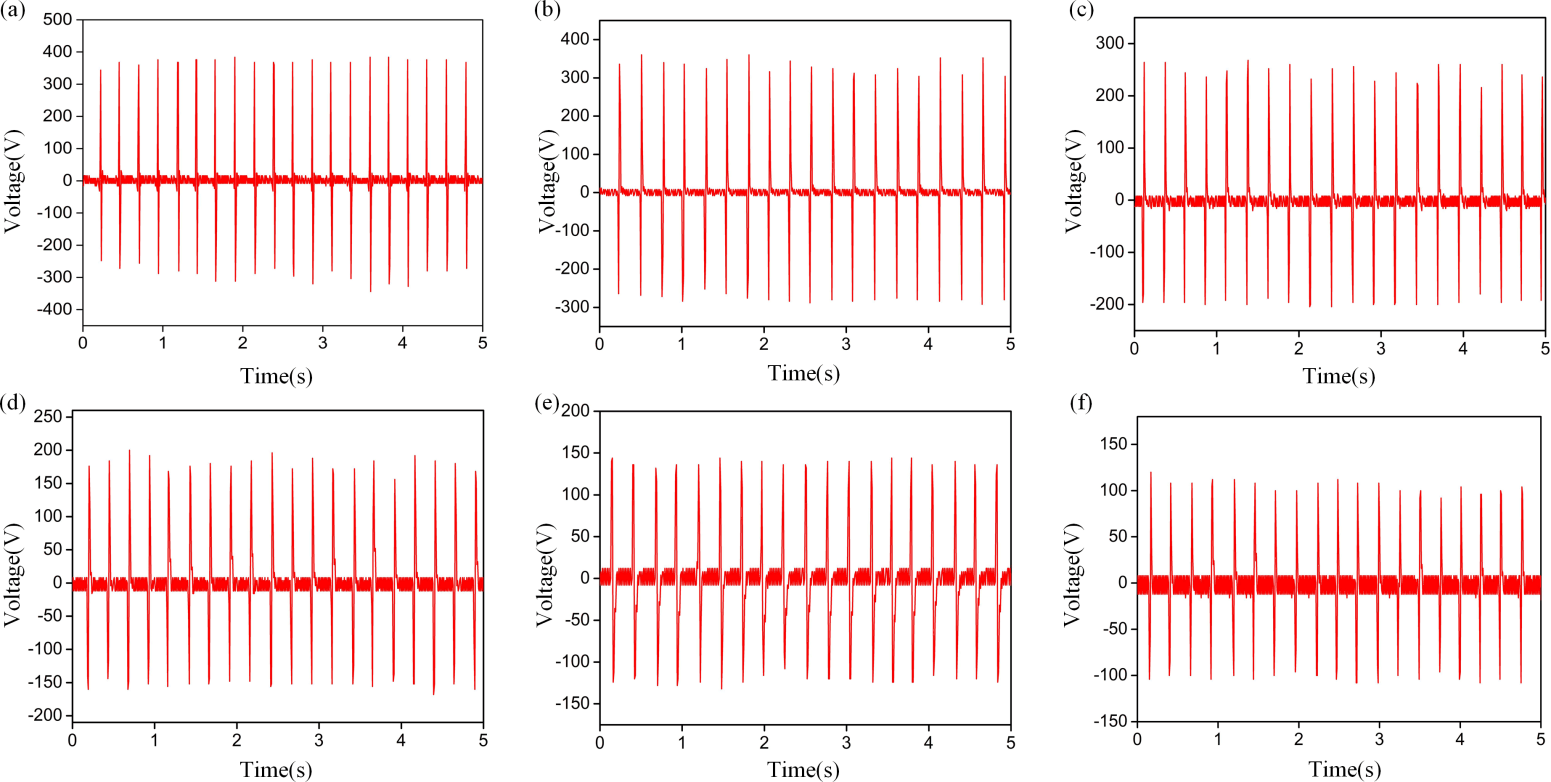
The output voltage sensor feedback at different humidity levels: 40% (a), 50% (b), 60% (c), 70% (d), 80% (e) and 90% (f).

**Figure 5:**
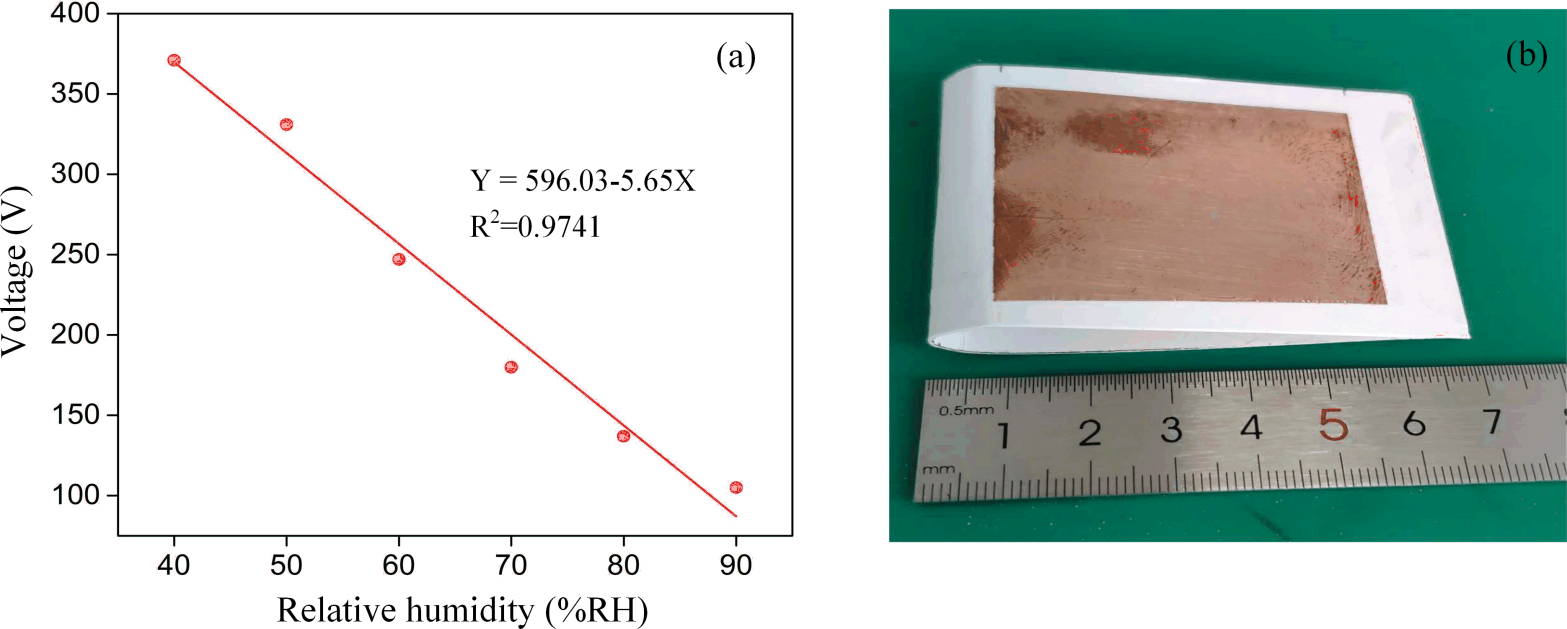
(a) The output voltage of the TVB-TENG as a function of RH (40–90%). (b) A representative photograph of the TVB-TENG.

The TENG output is affected by the humidity due to the triboelectric effect, and the preservation of the triboelectric charge at the surface is severely decreased by the humid environment [47]. It should be considered that the experiments with the TVB-TENG were performed in Chongqing, located in the southern region of China where it rains often and the annual relative humidity is usually above 40%. Thus, the humidity sensor response is limited due to a change in RH from 40 to 90% (Figure 4a–f). The dynamic change between the output voltage and the RH can be derived from the 2D graph. As the RH increased, there was a declining trend in the output signal. Figure 4 shows the output voltage of the TVB-TENG upon a change in RH. The output voltages of 371 V, 331 V, 247 V, 180 V, 137 V and 105 V corresponded to RH levels of 40%, 50%, 60%, 70%, 80% and 90%, respectively. As illustrated in Figure 5a, the output voltage as a function of RH has a linear fitting and the fitting equation for the output voltage (*Y*) and the relative humidity (*X*) can be represented as *Y* = 596.03 − 5.65*X*, with an *R*-squared value of 0.9741. A representative photograph of the TVB-TENG is shown in Figure 5b. Table 1 presents the humidity-sensing characteristics of the proposed humidity sensor in comparison with previously published studies [42–44,48–49]. The response and measurement range of the manufactured sensor are comparable to the sensor made from collagen–cotton fabric via the freeze-dried method [43].

**Table 1:** The results presented in this work are compared with previous studies for humidity detection.

Sensor type	Sensing material	Fabrication method	Measurement range	Response	Ref.

voltage-type	Ga-doped ZnO	hydrothermal method	45–80% RH	358	[42]
current-type	collagen–cotton fabric	freeze-dried method	50–90% RH	0.1287 μA/%RH	[43]
voltage-type	SnS₂/rGO	screen-printing method	0–97% RH	65	[44]
voltage-type	Al-doped ZnO	seed-assisted wet-chemical method	15–60% RH	1522	[48]
voltage-type	Fe-doped ZnO	seed-assisted wet-chemical method	5–65% RH	305	[49]
voltage-type	vitamin B powder	powder machine method	40–90% RH	5.65 V/%RH	current work

**Figure 6:**
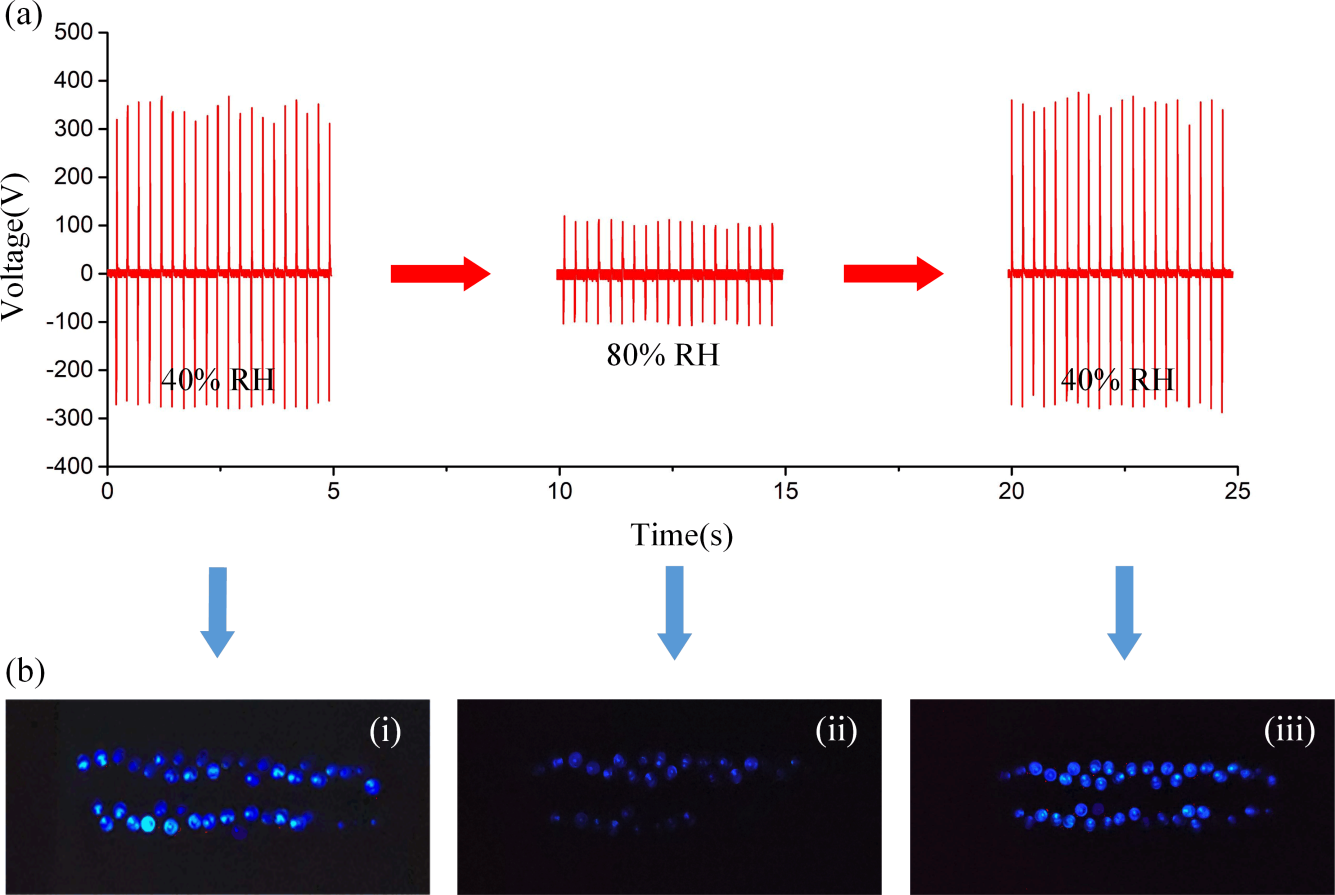
(a) Reversibility of a TVB-TENG-based humidity sensor. (b) The change in luminosity of thirty LEDs under different relative humidity conditions.

Furthermore, the reversibility of the TVB-TENG in terms of humidity sensing characteristics was also studied, as shown in Figure 6a. Thirty blue LEDs (according to the product specification of these 3 mm LEDs the voltage range is 1.8–2.2 V) were connected to the TENG humidity sensor as a real-time indicator of changes in the RH. The tests were conducted in environments with different RHs, and the luminosity of the LEDs was an indicator of changes in the RH. As illustrated in Figure 6b, when the surrounding humidity changes drastically, the change in the LED system luminosity can act as an alarm.

## Conclusion

This study proposed a novel TVB-TENG using vitamin B1 powder and Teflon tape. The fabricated device integrates a self-powered energy supply with a sensing system, and the change in relative humidity of the surrounding environment is properly detected. According to the experimental analysis, the results show that the TVB-TENG has a distinct humidity response. Further, the change in RH was also illustrated via the luminosity changes of the integrated LEDs. The device proposed in this study has great application potential in the area of environmental monitoring.
